# Advanced gynecologic malignancies treated with a combination of the VEGF inhibitor bevacizumab and the mTOR inhibitor temsirolimus

**DOI:** 10.18632/oncotarget.1834

**Published:** 2014-03-20

**Authors:** Sarina A. Piha-Paul, Jennifer J. Wheler, Siqing Fu, Charles Levenback, Karen Lu, Gerald S. Falchook, Aung Naing, David S. Hong, Apostolia M. Tsimberidou, Razelle Kurzrock

**Affiliations:** ^1^ Department of Investigational Cancer Therapeutics (Phase I Clinical Trials Program), University of Texas MD Anderson Cancer Center, Houston, TX; ^2^Department of Gynecologic Oncology, University of Texas MD Anderson Cancer Center, Houston, TX; ^3^Moores Cancer Center, The University of California San Diego, La Jolla, CA, U.S.A

**Keywords:** Gynecologic Malignancy, Bevacizumab, Temsirolimus

## Abstract

Background: Bevacizumab and temsirolimus are active agents in gynecologic tumors. Temsirolimus attenuates upregulation of HIF-1α levels, a resistance mechanism for antiangiogenics, and targets the PI3-kinase/AKT/mTOR axis, commonly aberrant in these tumors

Patients and Methods: We analyzed safety and responses in 41 patients with gynecologic cancers treated as part of a Phase I study of bevacizumab and temsirolimus.

Results: Median age of the 41 women was 60 years (range, 33-80 years); median number of prior systemic therapies was 4 (1-11). Grade 3 or 4 treatment-related toxicities included: thrombocytopenia (10%), mucositis (2%), hypertension (2%), hypercholesterolemia (2%), fatigue (7%), elevated aspartate aminotransferase (2%), and neutropenia (2%). Twenty-nine patients (71%) experienced no treatment-related toxicity greater than grade 2. Full FDA-approved doses of both drugs (bevacizumab 15mg/kg IV Q3weeks and temsirolimus 25mg IV weekly) were administered without dose-limiting toxicity. Eight patients (20%) achieved stable disease (SD) ≥ 6 months and 7 patients (17%), a partial response (PR) [total = 15/41 patients (37%)]. Eight of 13 patients (62%) with high-grade serous histology (ovarian or primary peritoneal) achieved SD ≥ 6 months/PR.

Conclusion: Bevacizumab and temsirolimus was well tolerated. Thirty-seven percent of heavily-pretreated patients achieved SD ≥ 6 months/PR, suggesting that this combination warrants further study.

## INTRODUCTION

Angiogenesis plays a key role in the pathogenesis of metastases, as new vessels provide growing tumors with nutrients, growth factors, oxygen, proteolytic enzymes, hemolytic factors, and hormones [1-3]. The vascular endothelial growth factor (VEGF) family of proteins and receptors is important in tumor angiogenesis and fundamental for tumor growth and metastasis [4, 5]. Bevacizumab is a monoclonal antibody specific for VEGF and prevents VEGF from interacting with its receptors on the surface of endothelial cells [6]. Bevacizumab inhibits angiogenesis, reducing tumor microvascularity and abrogating metastatic disease progression [6-8].

Diverse receptors in interconnected signaling pathways communicate with each other through cross-talk [9, 10]. Because communication is not restricted to a single receptor or signaling pathway, tumors frequently become resistant to antiangiogenic therapy through mechanisms such as upregulation of hypoxia-inducible factor (HIF)-1 α [3, 7, 11-21]. Adaptive responses to hypoxic conditions are modulated through HIF-1α over-expression, increasing levels of VEGF, which results in aggressive tumor growth and poor patient outcomes [3, 7, 11-21].

Temsirolimus is an inhibitor of mammalian target of rapamycin (mTOR), a serine/threonine kinase involved in initiating messenger ribonucleic acid (mRNA) translation [22, 23]. Aberrations of the phosphatidylinositol 3 (PI3)-kinase/AKT/mTOR pathway are common in several gynecologic malignancies such as endometrial and ovarian cancer [24, 25]. Renal cell carcinoma cell lines demonstrated inhibition of mTOR activity in i*n vitro* studies with temsirolimus, as well as reduced levels of HIF-1α, HIF-2α and VEGF [21]. Temsirolimus also inhibited VEGF production *in vitro* under both normoxic and hypoxic conditions through inhibition of HIF-1 expression and transcriptional activation in the human epidermal growth factor receptor (HER)-2 gene amplified breast cancer cell line BT474 [26].

Taken together, there are several compelling rationales for combining bevacizumab and temsirolimus in gynecologic tumors: i) temsirolimus inhibits mTOR and the PI3 kinase/AKT/mTOR pathway is critical in several gynecologic malignancies [24, 25]; ii) temsirolimus attenuates upregulation of HIF-1α levels, which may be a resistance mechanism for bevacizumab [21, 26]; iii) single-agent activity with temsirolimus and bevacizumab have been demonstrated in gynecologic cancers [27, 28]; and, iv) the two agents have non-overlapping toxicities. Here we report our experience treating patients with gynecologic malignancies with this combination therapy.

## RESULTS

### Demographic and Clinical Characteristics

Forty-one women with advanced, metastatic ovarian, uterine and cervical malignancies were enrolled starting in April 2008. Demographic and clinical characteristics are summarized in Table 1. The median age of patients was 60 years (range, 33-80 years). The most common cancer sites were ovarian followed by uterine. The median number of prior systemic therapies was 4 (range, 1-11). All patients had experienced disease progression on their prior therapy. No patients had received prior mTOR inhibitor therapy. Fourteen of forty-one patients (34%) had received prior therapy with bevacizumab. The median number of cycles (cycle = 21 days) completed for all patients was 4 (range, 1-25+). Thirty-four patients (83%) received more than 2 cycles. For patients with SD or better, the median number of cycles completed was 12 (range, 6-25+). At the time of analysis, three patients were continuing on therapy.

**Table 1:** Baseline Demographics and Clinical Characteristics

Characteristic	Total (%)
Number of patients	41
Age, years	
Median (Range)	60 (33-80)
Number of prior systemic therapies	
Median (Range)	4 (1-11)
ECOG performance status*	
0	10 (24)
1	25 (61)
2	6 (15)
Prior treatment	
Surgery	39 (95)
Radiation	18 (44)
Chemotherapy	41 (100)
Phase I trial	5 (12)
Temsirolimus	0 (0)
Bevacizumab	14 (34)
Primary Organ Site	
Fallopian Tube	1 (2)
Vagina	1 (2)
Ovarian	22 (54)
High grade serous^	13 (32)
Low grade serous	1 (2)
Endometroid#	2 (5)
Clear Cell	3 (7)
Transitional Cell	1 (2)
Undifferentiated	1 (2)
Carcinoma, Mullerian#	1 (2)
Uterus	11 (27)
Epithelial	9 (22)
Carcinosarcoma	1 (2)
Clear Cell	1 (2)
Cervix	6 (15)
Squamous	4 (10)
Adenocarcinoma	1 (2)
Neuroendocrine	1 (2)

* ECOG = Eastern Cooperative Oncology Group

^ includes two patients with peritoneal disease

# includes one patient with peritoneal disease

### Toxicity Assessment

Patients were enrolled in accordance with the planned 3+3 study design until dose level 11 (Table 2), at which point an expansion cohort for response (as described in the Methods section) was initiated. Dose escalation for the remaining two levels continued in accordance with the original dose escalation plan. Dose level 13 (bevacizumab 15 mg/kg and temsirolimus 25 mg) was reached and no MTD was obtained as we were able to reach the highest FDA-approved doses of both drugs (29).

**Table 2:** Dose-Escalation Schedule (21-day cycle), Grade 3/4 Toxicities* and Response

Dose Level	Temsirolimus IV on Days 1, 8 and 15	Bevacizumab IV on Day 1	SD≥6 months/PR Total Treated	Grade (G) 3/4 Toxicity (N)*
1-3	5 mg	5, 10 and 15 mg/kg	2/6	G3 HTN (1)^; G3 Hypercholesterolemia (1) All at 15 mg/kg of bevacizumab
4-6	12.5 mg	2.5, 7.5 and 15 mg/kg	2/3	G3 Neutropenia (1); G3 Elevated Aspartate Aminotransferase All at 15 mg/kg of bevacizumab
7-9	20 mg	2.5. 7.5 and 15 mg/kg	1/3	G3 Thrombocytopenia (1) at 15 mg/kg of bevacizumab
10-13	25 mg	2.5, 5, 10, 15 mg/kg	10/29	G4 Thrombocytopenia (1); G3 Fatigue (3)^; G3 Mucositis (1); G3 Thrombocytopenia (1); G4 Thrombocytopenia (1) All at 10 or 15 mg/kg bevacizumab

* Adverse events deemed at least possibly related to treatment were graded based on the Common Terminology Criteria for Adverse Events, version 3.0 (CTCAEv3.0)

^ was defined as a dose-limiting toxicity

Abbreviations: N, number of patients experiencing toxicity

**Table 3:** Tumor Molecular Analysis

Tumor Molecular Analysis		Response Comments
	Total (%)	
K-RAS Mutation		
Number Tested:	17	
Number with Mutation:	1 (6%)	KRAS mutation positive patient did not achieve SD ≥ 6 months/PR
N-RAS Mutation		
Number Tested:	17	
Number with Mutation:	1 (6%)	NRAS mutation positive patient did not achieve SD ≥ 6 months/PR
B-RAF Mutation		
Number Tested:	15	
Number with Mutation:	0	
PI3 Kinase Mutation		
Number Tested:	25	
Number with Mutation:	1 (4%)	PI3 Kinase mutation positive patient achieved a PR
PTEN Loss		
Number Tested:	2	
Number with Loss:	0	

Abbreviations: PR, partial response; SD, stable disease

All 41 patients with an advanced gynecologic malignancy experienced at least one adverse event that was possibly drug related. These events were mostly grade 1 or grade 2 and reversible. In fact, 29 patients (71%) experienced no treatment-related toxicity greater than grade 2. Grade 3 or 4 toxicities were as follows: thrombocytopenia (10%), mucositis (2%), hypertension (2%), hypercholesterolemia (2%), fatigue (7%), elevated aspartate aminotransferase (2%), and decreased absolute neutrophil count/leucopenia (2%). Among this subset of patients, two DLTs occurred (grade 3 hypertension at dose level 3 (bevacizumab 15 mg/kg and temsirolimus 5 mg) and grade 3 fatigue at dose level 13 (bevacizumab 15 mg/kg and temsirolimus 25 mg)) (Table 2). These toxicities were reversible when the dose was lowered or held. Of the 25 patients treated at the MTD, only 3/25 (12%) were dose-reduced for toxicities occurring during the first cycle. In these three instances, the temsirolimus was dose reduced from 25 to 20 mg. The causes of dose reduction were grade 3 fatigue (n=1), grade 2 mucositis (n=1) and grade 2 diarrhea (n=1).

There were no gastrointestinal perforations, thromboembolic events or cases of significant proteinuria. Two patients experienced gastrointestinal-vaginal fistula. One patient had vaginal cancer (adenocarcinoma) and had received prior pelvic radiation. She had a history of gastrointestinal-vaginal fistula prior to protocol entry and had been surgically diverted with colostomy. While on therapy, she developed worsening vaginal discharge and perineal pain. Though there was no radiographic evidence on computed-tomography (CT) scans of the abdomen/pelvis of fistula, clinically she was believed to have gastrointestinal-vaginal fistula (grade 2). She completed only one cycle of therapy before withdrawing consent. The second patient had low grade serous ovarian cancer with no prior history of pelvic radiation. She was found on CT of the abdomen and pelvis to have gastrointestinal-vaginal fistula (grade 1). She was taken off protocol for fistula formation and received only one cycle of therapy. Finally there was one patient who developed wound healing complications. This patient had cervical cancer (squamous cell) and a history of prior radiation. She had undergone vaginoplasty with subsequent development of recto-vaginal fistula and necrotic tumor in the vagina. Her vaginal mass decreased in size by 28% per RECIST on the first restaging but her course was complicated by persistent perirectal abscess treated with oral antibiotics. The abscess remained stable during her three cycle of treatment; however, the patient was taken off protocol as she wished to pursue elective colostomy.

### Antitumor Activity

**Figure 1:**
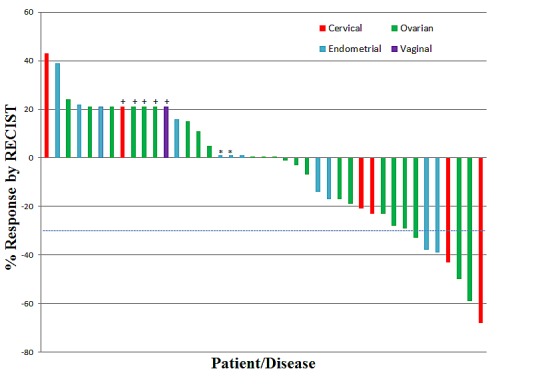
Waterfall Plot Depicting Best RECIST Response by Patient Individual patients/disease sites are represented by vertical bars on the X-axis. The best RECIST response (%) is depicted on the Y-axis. Thirty-four of the 41 patients were measurable by RECIST. Five patients were assigned a value of +21% for clinical progression or new lesions (+). Two patients were assigned a value of +1% as they were evaluable but not measurable by RECIST and had stable disease (*). Dotted line shows 30% response by RECIST.

Thirty-four of the 41 patients had disease that was measurable by RECIST and reached restaging. All patients, however, were considered evaluable. Figure 1 is a waterfall plot depicting best response by patient. Five patients were assigned an arbitrary value of +21% for early clinical progression or new lesions upon restaging. The remaining two patients were assigned a value of +1% as they were evaluable but not measurable by RECIST and had SD. Seven patients (17%) achieved a partial response (PR). SD lasting ≥ 6 months was observed in 8 patients (20%). The total SD ≥ 6 months/PR rate was 15/41 patients (37%). Details regarding these patients including dose level, duration of treatment and best response by RECIST are described in Table 4.

**Table 4:** Stable Disease > 6 months or Partial Response (PR) by RECIST and Characterization by Patient

Disease Site	Histology	Dose Level	Best Response	# of Prior Cytotoxic Regimens	Duration of Treatment (weeks)	PTEN*	PI3K Mutation	RAS Mutation	RAF Mutation
Uterus	Clear Cell Carcinoma	5	-39%	3	36	ND	Y	ND	N
Ovary	High Grade Serous	13	-33%	4	27	ND	N	N	N
Cervix	Squamous	6	-43%	3	18	ND	N	ND	ND
Ovary	High Grade Serous	13	-59%	7	63+	ND	N	ND	N
Uterus	Epithelial	13	-38%	4	21	P	N	N	ND
Ovary	High Grade Serous	13	-50%	2	48	ND	ND	ND	ND
Cervix	Squamous	13	-68%	1	60+	ND	ND	ND	ND
Peritoneum	High Grade Serous	2	0%	4	24	ND	N	ND	ND
Ovary	High Grade Serous	3	-7%	5	36	ND	N	ND	ND
Uterus	Epithelial	8	Non-measurable by RECIST	1	36	ND	N	N	ND
Ovary	High Grade Serous	12	-28%	4	36	ND	ND	ND	ND
Fallopian Tube	Epithelial	13	-17%	3	36	ND	N	N	N
Peritoneum	High Grade Serous	13	-19%	5	30	ND	N	N	N
Uterus	Epithelial	13	Non-measurable by RECIST	2	75+	P	ND	N	N
Ovary	High Grade Serous	13	-29%	6	24	ND	ND	ND	ND

Abbreviations: ND, not done; N, no; Y, yes; P, present

* PTEN is tested by immunohistochemistry; absence of PTEN generally indicates an aberration

The only patient with clear cell carcinoma of the uterus achieved a PR. Of 11 patients with cancer arising from the uterus, four (36%) had SD ≥ 6 months or a PR (these included 3/9 patients with epithelial uterine cancer and 1/1 patient with clear cell carcinoma). Among 11 patients with high-grade serous ovarian cancer, three (27%) achieved a PR and three (27%) attained prolonged SD ≥ 6 months (total = 6/11 (55%) with SD ≥ 6 months/PR). Among four patients with primary peritoneal disease, the two patients with high grade serous histology achieved SD ≥ 6 months. Among four patients with squamous cell carcinoma of the cervix, two (50%) achieved a PR. Finally, one patient with fallopian tube cancer achieved prolonged SD (≥ 6 months). Characteristics of the responders are detailed in Table 5.

**Table 5:** Response Data by Disease Site and Histology

Disease Site/Histology	# of patients treated	PR	SD≥ 6 months	Median Number of cycles completed (range)
Uterus	11			6 (2-25+)
Epithelial	9	1	2	5 (2-25+)
Carcinosarcoma	1			6
Clear Cell	1	1		12
Ovary	18			4 (1-21+)
High grade serous	11	3	3	8 (1-21+)
Low grade serous	1			1
Endometroid	1			2
Clear Cell	3			4 (3-5)
Transitional Cell	1			1
Undifferentiated	1			4
Peritoneum	4			4.5 (1-10)
High grade serous	2		2	9 (8-10)
Carcinoma, Mullerian	1			1
Endometroid	1			1
Cervix	6			4.5 (1-20+)
Squamous	4	2		4.5 (1-20+)
Adenocarcinoma	1			6
Neuroendocrine	1			2
Fallopian Tube	1			
Epithelial	1		1	12
Vagina	1			
Adenocarcinoma	1			1

There was no obvious dose-response relationship. Three of 7 patients (43%) treated on dose levels 1 through 5 achieved SD≥6 months/PR versus 12 of 34 patients (35%) on dose levels 6 through 13 (P = 0.7). Six of 16 patients (38%) at dose levels 1 through 12 achieved SD≥6 months/PR versus 9 of 25 patients (36%) at dose level 13 (p = 1).

### Molecular Analysis and Association with Response

When archival cell blocks for patients were available, CLIA-certified testing was performed for BRAF, NRAS, KRAS, and PI3 kinase mutations along with evaluation for PTEN loss. PI3 kinase mutational status was known for 25/41 of patients (61%) and was positive in one (45%). This patient achieved a PR. PTEN status was known for 2/41 (5%), and PTEN loss was identified in none of the patients tested. For BRAF, mutational status was evaluated in 15/41 (37%) and was positive in none of the patients tested. Finally, KRAS/NRAS mutations were evaluated in 17/41 of the patients (41%). One patient (6%) had an NRAS mutation and 1 (6%) patient had a KRAS mutation. Neither of these patients achieved SD ≥ 6 months or a PR (Table 3). Among the 15 patients with a SD ≥ 6 months/PR, 10 patients (67%) were tested for PI3 kinase mutations and only one was positive. Two patients were tested for PTEN loss (one of whom was also tested for PIK3CA mutation) and neither was positive.

## DISCUSSION

There is a paucity of definitive data regarding optimal therapy in patients with advanced gynecologic malignancies after failure of first-line agents. Many of these patients are heavily pretreated, and thus less likely to tolerate full-dose cytotoxic therapy. In our study, treatment with bevacizumab and temsirolimus showed excellent tolerance even when the maximum FDA-approved dose of each drug was used in the combination. Twenty-nine patients (71%) experienced no treatment-related toxicity greater than grade 2 and only two DLTs were observed. A minority of patients (n=12) experienced grade 3 or 4 toxicities, most of which were cytopenias, dyslipidemia, elevated liver function tests (AST) or fatigue, and attributed to temsirolimus. These toxicities were reversible with discontinuation of treatment or lowering of the dose, and were mostly managed with supportive care while patients were on therapy. Further, we had no complications of gastrointestinal perforation, significant proteinuria or thromboembolic events. One patient developed a new gastro-vaginal fistula on therapy. One patient with wound healing complications from prior vaginoplasty and locally advanced necrotic vaginal tumor, had shrinkage of her vaginal tumor (-28%) at first restaging and was taken off study because she wished to proceed with elective colostomy.

Moroney et al [39] recently reported a SD ≥ 6 months/PR/CR rate of 38% in patients with advanced gynecologic and breast malignancy treated with liposomal doxorubicin, bevacizumab and temsirolimus. Our SD ≥ 6 months/PR rate was 37% which is comparable without the added toxicity of liposomal doxorubicin. Our patients, like those reported by Moroney et al [39], were heavily pretreated with a median of four prior systemic therapies. However, 51/74 patients (69%) of the patients treated by Moroney et al [39] experienced any tumor regression, while only 18/41 patients (44%) of our patients did. Only a randomized study would be able to answer the question as to whether or not liposomal doxorubicin adds activity to the bevacizumab and temsirolimus regimen.

Mutations in PI3 kinase result in activation of the PI3 kinase/AKT/mTOR pathway and are present in various tumor types [40, 41]. In gynecologic malignancies, phosphoinositide-3-kinase, catalytic, alpha polypeptide (*PIK3CA*) mutations are found in 23%, 10% and 12% of endometrial, ovarian, and cervical cancers, respectively[42]. PTEN typically acts as a repressor of the PI3 kinase/AKT/mTOR pathway and its loss results in its constitutive activation [43]. PTEN mutations are found in 40%, 4%, and 5% of endometrial, ovarian, and cervical cancers, respectively [42]. We previously reported a PR rate of 35% in heavily pretreated patients with diverse cancers and somatic PI3 kinase mutations treated with PI3kinase/AKT/mTOR pathway inhibitors [44]. In addition, Janku and colleagues [45] also showed a PR rate of 30% in patients with breast and gynecologic malignancies harboring PIK3CA mutations and treated with relevant pathway inhibitors. The relationship between mutational status and response in our patients is unclear because of the limited numbers of patients with tissue available for testing. Twenty-five of 41 patients (61%) were analyzed for *PIK3CA* mutation and only one was positive. This patient achieved a PR. Of the 24 patients who were negative for *PIK3CA* mutation, 9 patients (38%) achieved SD ≥ 6 months/PR. Further, of the 15 patients who achieved SD ≥ 6 months/PR, only 10 had a known *PIK3CA* mutation status. While these results suggest that *PIK3CA* mutations are not necessary to achieve SD ≥ 6 months/PR, there are several limitations to this observation. For example, our laboratory only evaluated exons 9 and 20 at the time of patient testing. These exons code only for the helical and kinase functional domains of *PIK3CA*, respectively. Mutations in the p85 binding site, RAS binding site and C2 region were not assessed so that other PI3 kinase mutations may have been missed. Further, aberrations in mTOR and AKT, though known to exist in cancer [46-48], were not assessed. Finally, PTEN loss and/or mutation are common in endometrial cancers [49] and merit more extensive evaluation. Unfortunately, limited tissue availability resulted in PTEN IHC testing being evaluated in only a small number of our patients. Of interest in this regard, Moroney et al [39] demonstrated that 13 of 25 patients (52%) with gynecologic malignancies that had *PIK3CA* mutations or PTEN loss and were treated with liposomal doxorubicin, bevacizumab, and temsirolimus achieved SD ≥ 6 months/PR/CR. Further, the combination of bevacizumab and temsirolimus has shown preliminary evidence of activity in other tumors in which activation of the *PI3 kinase*/AKT/mTOR axis has been implicated, including salivary duct tumors [50] and lymphangioleiomyomatosis [51].

Other mechanisms for response could be operative in our patients. For example, bevacizumab has antiangiogenic properties, and resistance to this agent is caused in part by upregulation of HIF-1α [3, 7, 11-21]. Further, temsirolimus abrogates HIF-1α mRNA transcription and this may drive response in some patients [26].

There are several limitations to this study. First, molecular analysis could not be performed in many of the patients because of lack of tissue and therefore a biomarker was not elucidated. Second, these patients were heavily pretreated, with a median of four prior systemic therapies, and this may have limited response signals. Third, patients were treated on a variety of dose levels. However, in relation to the latter point, it should be noted that there was no obvious dose-response relationship, with SD≥6 months/PR attained even at dose levels 1 and 2, and no difference in rate of SD≥6 months/PR in patients treated at the highest dose level (9 of 25 patients (36%)) versus those treated at lower dose levels (6 of 16 (38%)) (p = 1). However, the study was not designed to answer the dose-response question in a definitive manner.

In conclusion, the combination of bevacizumab and temsirolimus was well tolerated in our study and has demonstrated clinical activity in patients with advanced gynecologic malignancy having undergone extensive prior therapy. The overall rate of SD ≥ 6 months/PR was 37%, and 8 of 13 patients (62%) with high-grade serous histology (ovarian or primary peritoneal) achieved SD ≥ 6 months/PR. Further study of this combination in larger populations with gynecologic cancers is warranted.

## PATIENTS AND METHODS

### Study Design and Dosing

The experience with gynecologic malignancies reported is part of a single institution, phase I, open-label, dose-escalation study. This trial was open to all patients with advanced or metastatic cancer refractory to standard therapy, relapsed after standard therapy, or who had no standard therapy available that improves survival by at least three months. (The trial successfully completed dose escalation to the highest specified dose level, that is dose level 13, which consisted of the highest FDA-approved doses of both drugs (bevacizumab 15 mg/kg IV every 3 weeks and temsirolimus 25 mg IV weekly)[29].)

Treatment was administered on an outpatient basis at MD Anderson Cancer Center. A cycle of therapy was 21 days. No investigational or commercial agents or therapies other than those described here could be administered with the intent to treat the patient's malignancy. Bevacizumab was given on day 1 only of each cycle while temsirolimus was given weekly on days 1, 8 and 15 (Table 2). Restaging scans were performed after every two cycles. Consent was obtained and patients were treated in accordance with MD Anderson Cancer Center Institutional Review Board guidelines.

The protocol followed a standard 3+3 design. If one patient in a cohort experienced a DLT during the first cycle, three additional patients were enrolled and treated at that dose level. If at any time more than 33% of patients in a cohort experienced a DLT, that cohort was closed to additional patients. Adverse events were graded based on the Common Terminology Criteria for Adverse Events, version 3.0 (CTCAEv3.0) [30]. DLTs were defined as any grade three or four non-hematologic toxicity that was possibly, probably or definitely related to any of the study medications, with the following exceptions: a) any grade four hematologic toxicity lasting less than two weeks, and b) any grade four nausea or vomiting lasting less than five days. DLTs had to occur within the first cycle of treatment. Of note, early in the trial multiple significant responses were observed. If a response was observed in a particular tumor type with the study drug combination, expanded enrollment was permitted for up to a total of 14 patients with that tumor type at the highest dose level deemed safe at the time of patient enrollment. All enrolled participants with that tumor type were considered in the DLT analysis. For the purpose of dose expansion, a tumor response was defined as one or more of the following: 1) stable disease for more than or equal to four months (SD ≥ 4 months), 2) decrease in the sum of target lesions by more than or equal to 20% by RECIST criteria 1.0, or 3) decrease in tumor markers by more than or equal to 25%. This resulted in cohort expansions in gynecologic malignancies.

### Eligibility Criteria

Key inclusion criteria were histologically-documented, advanced or metastatic solid tumors refractory to standard treatment or for which no standard therapy was available; Eastern Cooperative Oncology Group (ECOG) performance status ≤ two; absolute neutrophil count ≥ 1 x 10^9^/L; platelet count ≥ 50.0 x 10^9^/L; serum creatinine < 3.0 mg/dL, alanine transferase (ALT) ≤ five times the upper limit of normal (ULN); bilirubin ≤ 3.0 mg/dL, total cholesterol < 350 mg/dL; and, triglyceride <400 mg/dL. Key exclusion criteria were clinically significant, unexplained bleeding or hemoptysis within 28 days prior to study entry; poorly controlled hypertension (systolic blood pressure ≥ 140 mm Hg, diastolic pressure ≥ 90 mm Hg); patients with clinically significant cardiovascular disease; and, pregnancy. Prior exposure to mTOR and VEGF-inhibitors were not exclusion criteria for study entry, nor were patients with a history of venous thromboembolism excluded.

### Assessment of Tumor Response

Tumor measurements were performed on patients with measurable disease pre-treatment and every two cycles thereafter. Measurable target lesions were evaluated for response using Response Evaluation Criteria in Solid Tumors (RECIST 1.0) [31, 32]. For purposes of this report, prolonged stable disease (SD) was defined as lasting ≥ 6 months.

### Molecular Analysis (PIK3CA, BRAF, NRAS and KRAS, BRAF and PTEN)

*PIK3CA*, *BRAF*, *NRAS* and *KRAS* mutations were investigated in archival formalin-fixed, paraffin-embedded tissue blocks. DNA was extracted from microdissected paraffin-embedded tumor sections and analyzed using a polymerase chain reaction (PCR)-based DNA sequencing method for *PIK3CA* mutations in codons [c]532-554 of exon 9 (helical domain) and c1011-1062 of exon 20 (kinase domain)[33], which included the mutation hot spot region of the *PIK3CA* proto-oncogene by Sanger sequencing following amplification of 276 bp and 198 bp amplicons, respectively. Codons 12, 13, and 61 were examined for *KRAS* and *NRAS* mutations and for *BRAF, codons* 468-474, codons 595-600, and mutations of exon 15 by pyro-sequencing were examined as previously described [34]. PTEN loss by IHC generally indicates aberrant or mutated PTEN, which serves to activate the PI3 kinase/AKT/mTOR pathway [35-38]. Formalin-fixed paraffin-embedded sections (5 µm thick) from biopsy or resection specimens were used for IHC analysis. The sections were stained with antibody to PTEN (Dako, Carpinteria, CA). All histologies were centrally reviewed and all testing was performed in the Clinical Laboratory Improvement Amendment (CLIA) –certified Molecular Diagnostic Laboratory (MDL) at MD Anderson.
